# Effect of Environmental Complexity and Stocking Density on Fear and Anxiety in Broiler Chickens

**DOI:** 10.3390/ani11082383

**Published:** 2021-08-12

**Authors:** Mallory G. Anderson, Andrew M. Campbell, Andrew Crump, Gareth Arnott, Ruth C. Newberry, Leonie Jacobs

**Affiliations:** 1 Department of Animal and Poultry Sciences, Virginia Tech, Blacksburg, VA 24061, USA; mallory8@vt.edu (M.G.A.); drewc1@vt.edu (A.M.C.); 2 Centre for Philosophy of Natural and Social Science, London School of Economics and Political Science, London WC2A 2AE, UK; andrewcrump94@gmail.com; 3 School of Biological Sciences, Queen’s University Belfast, Belfast BT9 5DL, UK; g.arnott@qub.ac.uk; 4 Department of Animal and Aquacultural Sciences, Faculty of Biosciences, Norwegian University of Life Sciences, 1432 Ås, Norway; ruth.newberry@nmbu.no

**Keywords:** broiler chicken, affective state, environmental complexity, stocking density, anxiety, fear, animal welfare, attention bias, tonic immobility

## Abstract

**Simple Summary:**

Broiler chickens are conventionally housed in monotonous environments at high stocking densities, which can negatively affect their welfare. This study evaluated the impact of environmental complexity and stocking density on anxiety and fear in broilers. Through behavioral testing, we found that broilers housed at higher densities responded less fearfully than those housed at the lower density, which is contradicting to expectations and previous research. Broilers housed in complex environments exhibited responses consistent with reduced anxiety compared to broilers housed in monotonous environments, suggesting improved welfare for broilers housed in the complex environment.

**Abstract:**

Barren housing and high stocking densities may contribute to negative affective states in broiler chickens, reducing their welfare. We investigated the effects of environmental complexity and stocking density on broilers’ attention bias (measure of anxiety) and tonic immobility (measure of fear). In Experiment 1, individual birds were tested for attention bias (*n* = 60) and in Experiment 2, groups of three birds were tested (*n* = 144). Tonic immobility testing was performed on days 12 and 26 (*n* = 36) in Experiment 1, and on day 19 (*n* = 72) in Experiment 2. In Experiment 1, no differences were observed in the attention bias test. In Experiment 2, birds from high-complexity pens began feeding faster and more birds resumed feeding than from low-complexity pens following playback of an alarm call, suggesting that birds housed in the complex environment were less anxious. Furthermore, birds housed in high-density or high-complexity pens had shorter tonic immobility durations on day 12 compared to day 26 in Experiment 1. In Experiment 2, birds from high-density pens had shorter tonic immobility durations than birds housed in low-density pens, which is contrary to expectations. Our results suggest that birds at 3 weeks of age were less fearful under high stocking density conditions than low density conditions. In addition, results indicated that the complex environment improved welfare of broilers through reduced anxiety.

## 1. Introduction

Environmental enrichment can be defined as “a modification of the environment of captive animals, thereby increasing the animal’s behavioral possibilities and leading to improvements of their biological function” [1]. Although results vary depending on the outcome variables assessed, the addition of different structures to the environment adds complexity and can have enriching effects for livestock, including broiler chickens [2,3,4]. These provisions are therefore typically referred to as enrichments.

Fear and anxiety raise welfare concerns because they generate negative affect and, if chronically aroused, highlight an animal’s inability to cope with its environment [5,6]. Fear is a short-term emotional response motivating flight from, or freezing in response to, a currently present, immediate threat to survival, while anxiety is a longer-term emotional response motivating vigilance (i.e., alertness) in response to perceived potential threat and is amplified by adverse pre-and postnatal life experiences [5,7,8,9,10]. These systems have evolved as adaptive mechanisms promoting survival in dangerous situations through temporary activation of sympathetic and hypothalamic-pituitary-adrenal axis activity and suspension of growth-promoting parasympathetic activity [5]. However, excessive fear in broilers can be maladaptive, provoking panicked escape behaviors that cause injury, pain, and suffocation [11]. In addition, high levels of fear and anxiety impair the birds’ ability to cope with environmental change, such as handling, transport, and loud noises, and have been linked with a worsened feed conversion ratio [12,13]. In many studies, fear in birds is measured using a tonic immobility (TI) test. TI is an anti-predator freezing response (feigning death) which prey species exhibit as a last resort when captured [14]. Longer TI durations have revealed higher levels of fear in broilers handled roughly compared to gently [15], manually caught compared to mechanically caught [16], or heat-stressed [12] or shocked [17] prior to testing compared to control. A TI test could provide valuable insight into broiler fear levels when handled after rearing in environments varying in complexity and stocking density.

Level of anxiety can be evaluated through an attention bias (AB) test. AB describes the differential, affect-mediated allocation of attention towards one stimulus compared to others [18]. In particular, anxious (vigilance) affective states can increase AB towards a stimulus [18]. Humans with clinical anxiety show a greater AB towards threatening stimuli than those without anxiety [19,20,21], and studies involving macaques [22], sheep [6,23], cattle [24], and laying hens [25] have validated AB testing as a measure of anxiety level, where animals receiving an anxiogenic drug spent more time looking towards a threatening stimulus and showed increased vigilance behavior compared to control animals. For example, after receiving an anxiogenic drug, laying hens exposed to a conspecific alarm call were slower to feed, faster to vocalize, and exhibited increased locomotion, compared to hens that received a saline injection [25]. These findings suggest that relatively anxious hens allocate more attention to a perceived threat, suggesting that this test could possibly serve as a tool to measure anxiety levels in broilers also. Although studies have reported successful differentiation of AB in animals, others have found unexpected or null results [26,27,28]. To our knowledge, however, AB in broilers has not been previously tested.

Typical broiler chicken housing lacks complexity, such as provision of perches or preferred dustbathing substrate, limiting the expression of diverse natural behaviors, potentially contributing negatively to broiler welfare and performance [1,29,30,31,32]. High stocking density is another welfare concern in broilers. For instance, high stocking densities can lead to poor foot health [3,11,33] and may increase fear (response to a detected threat) [5]. Lack of environmental complexity has also been associated with fear in broilers [11]. However, behavioral indices of fear were not affected when birds were housed with or without access to string or barrier perches at various stocking densities [34,35,36], raising questions about how stocking density affects fearfulness of broilers housed in a complex environment.

A reported benefit of adding perches as an enrichment for broilers is that the birds were less aggressive and experienced fewer disturbances while resting compared to broilers without perches [29,35]. For broilers, low perching platforms are used more than single linear perches, probably because heavy birds find them easier to balance on [37], and they were found to reduce avoidance of people, suggesting they reduced fear [38]. Moreover, while broilers are conventionally provided with a single type of litter over the whole floor, adding additional substrate materials can be enriching given that they vary in their value for different functions. For example, sand has been found to increase dustbathing behavior and activity levels compared to rice hull, paper, or wood shaving substrates [39], and adding maize roughage increased foraging behavior compared to wood shavings alone [32]. In addition, broilers housed with novel objects exhibited shorter durations of tonic immobility following acute stressors (sound, heat, and crating stress) compared to the control (no added objects), indicating decreased fearfulness [40]. Given this evidence, increasing environmental complexity with perches, sand, and novel objects would enhance broiler welfare through reduced anxiety and fearfulness.

Potential combined effects of environmental complexity and stocking density on fear and anxiety in broilers have not previously been examined experimentally. Our objective was to investigate the impact of complex housing conditions and stocking density on fearfulness, as measured through a TI test, and anxiety, using an AB test. We hypothesized that broilers housed in a high-complexity, low-density environment would experience the lowest levels of fear and anxiety, whereas broilers from a low-complexity, high-density environment would experience the highest levels of fear and anxiety, with a low-complexity, low-density environment and a high-complexity, high-density environment showing intermediate results. In particular, we predicted that higher levels of fear and anxiety would be reflected by longer TI durations and stronger AB to perceived threatening stimuli.

## 2. Materials and Methods

### 2.1. Birds, Treatments, and Housing

Two experiments were conducted. In each, 1620 male Ross 708 chicks (total *n* = 3240), vaccinated against Marek’s disease, were obtained at day 0 from a commercial hatchery (Elizabethtown, PA, USA). Upon arrival to the research facility, chicks were randomly allocated to one of four treatment groups in a 2 × 2 factorial design with environmental complexity (low-complexity (LC) vs. high-complexity (HC)) and stocking density (low-density (LD) vs. high-density (HD)) as factors at pen level. Each treatment group was replicated three times (12 pens in total), distributed in a randomized complete block design.

All pens (14.5 m^2^) contained standard pine shavings as bedding (approximately 10 cm depth), four hanging galvanized tube feeders (~12 kg capacity; no longer in production, but similar to “Flex” chicken feeder unit, SKU# CO30131, Hog Slat, Newton Grove, NC, USA), and three water lines (Valco Industries, Inc., New Holland, PA, USA), each with three nipple drinkers. All birds had ad libitum access to water and commercially-formulated broiler chicken feed (starter day 0–14, grower day 15–28, and finisher day 29–50). The birds were fed a corn/soy-based diet which met their nutritional requirements [41]. Birds had access to three heat lamps/pen and 24 h light in the first 7 days, followed by a light:dark schedule of 18L:6D, with a light intensity of approximately 15 lux during light hours. Due to a technical issue in Experiment 1, birds received 24 h light for 7 additional days during week 2 of age. House temperature was gradually decreased from 35 °C on day 1 to 21 °C on day 50 by assessing bird comfort. Comfort was evaluated based on behaviors indicative of heat or cold stress (panting or huddling respectively), bird activity (birds are active and alert when a person enters the facility), and bird distribution (birds are showing a somewhat homogenous distribution throughout the pen). In Experiment 1, all birds received a therapeutic dose of antibiotics via the water lines from day 33–40 in response to a pathogen exposure.

### 2.2. Environmental Complexity

**Figure 1:**
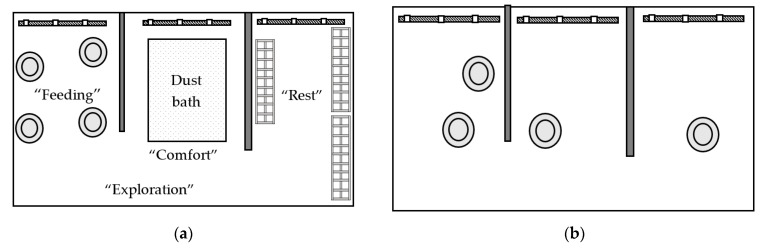
(**a**) High-complexity pen with four functional spaces for “feeding”, “comfort”, “resting”, and “exploration”. The feeding space contained four feeders (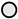) and pecking stones, the “comfort” space included a sand dust bath (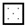), the resting space contained three perches (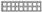), and the exploration space contained varying pairs of enrichment objects. The feeding, comfort, and resting spaces each contained a water line with three nipple drinkers (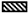). (**b**) Low-complexity (control) pen, containing four feeders and three water lines.

**Figure 2:**
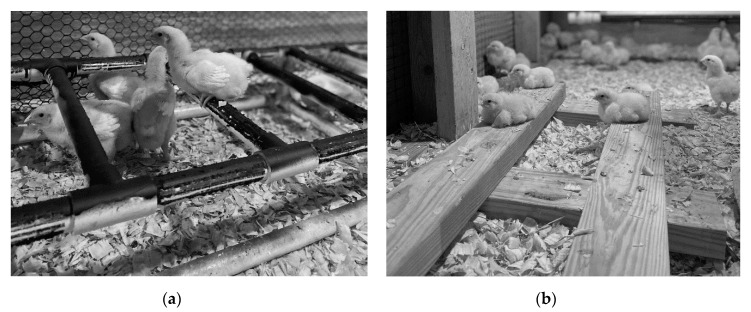
Photograph of the perch design in high-complexity pens in (**a**) Experiment 1 (*n* = 3/pen) and (**b**) Experiment 2 (*n* = 3/pen).

HC pens contained four functional spaces (Figure 1a), including space for “feeding” (approximately 3 m^2^), “comfort” (approximately 3 m^2^), “resting” (approximately 3 m^2^), and “exploration” (approximately 4.3 m^2^). The feeding, comfort, and resting spaces included a water line. The feeding space contained four feeders and one third of a medium PECKstone^TM^ (Proteka, Inc., Lucknow, ON, Canada) broken into smaller pieces. The comfort space contained a wooden-frame dust bath (180 cm L × 91 cm W × 10 cm H) filled with 68 kg of playground sand (QUIKRETE, Atlanta, GA, USA) that was raked and partially replaced when depleted. The resting space in Experiment 1 included three perches (182.9 cm L × 30.5 cm W × 8.5 cm H) constructed of 1.9 cm diameter PVC pipe, which was sprayed with textured black spray paint (Rust-Oleum, Vernon Hills, IL, USA) to enhance grip while perching (Figure 2a). Birds had access to 7.6 cm of linear perch space/bird in high-density pens, and 15.2 cm/bird in low-density pens. In Experiment 2, the PVC pipes were replaced with three wide wooden perches forming a platform (121.9 cm L × 45.7 cm W × 7.6 cm H; Figure 2b), providing 76 cm^2^ of space/bird in the low-density pens, and 39 cm^2^ of space/bird in the high-density pens. The exploration space contained a pair of enrichment objects, starting on day 2 of age. Six objects were randomly paired into three groups of two, combining a nutritional and an occupational enrichment object, and these pairs were rotated every three days according to a randomized schedule to maintain variation and novelty (Table 1). The LC pens had a similar set-up to the HC pens with four spaces, but without the peck stones, dust bath, perching platforms, or enrichment objects to differentiate the spaces into different functional areas (Figure 1b).

### 2.3. Stocking Density

The HD pens were stocked with 180 chicks/pen, resulting in 42.1 kg/m^2^ at day 50 in Experiment 1, and 42.6 kg/m^2^ in Experiment 2 (Table 2). The LD pens were stocked with 90 chicks/pen and reached a density of 23.8 kg/m^2^ at day 50 in Experiment 1, and 23.3 kg/m^2^ in Experiment 2 (Table 2).

### 2.4. Experiment 1—Attention Bias Test

**Figure 3:**
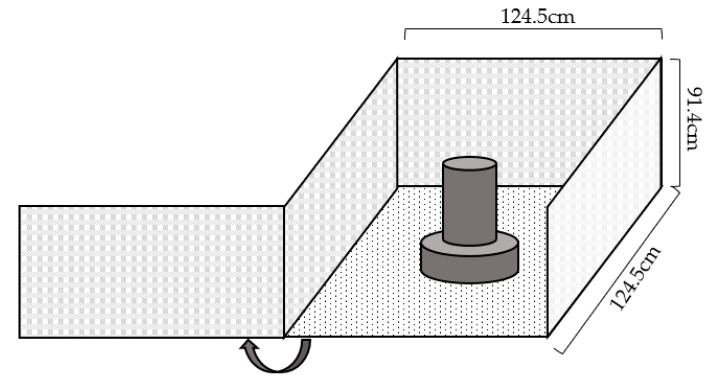
Diagram of the attention bias (AB) arena used in Experiments 1 and 2. A familiar feeder (exact same as provided in pens) was placed in the center of the arena and wood shavings were provided as litter.

A square testing arena was constructed with two plastic, perforated folding partitions (approximately 124.5 cm L × 124.5 cm W × 91.4 cm H) with pine shavings on the floor and a feeder containing commercial feed, oats, and mealworms (Figure 3). The arena was located in a separate room adjacent to, but separate from, the broilers’ home pens.

AB testing (modified from [18,25,42]) was performed with five randomly selected birds/pen (*n* = 60 birds across pens) on days 30, 32, and 33 of age. The testing order of pens was randomized. Each bird was tested separately by one observer, another person was present to move birds to and from the testing arena. The test started when the bird was placed in the AB arena. Immediately thereafter, an 8 second (s) conspecific alarm call was played from portable speakers (FUGOO, Van Nuys, Irvine, CA, USA) at full volume (95 dB). The alarm call was recorded from a chicken signaling a ground predator, which previous playback experiments have found to elicit a vigilance response [42]. Following the alarm call, latency to begin feeding was recorded. If the bird began feeding at any point during the test, it was allowed approximately 10 s to feed, then the alarm call was played a second time, and latency to resume feeding was recorded. The test ended when the bird resumed feeding a second time (maximum test duration of 300 s). Birds that never began feeding received a maximum latency to begin feeding score of 300 s and those failing to resume feeding received no score (missing data). Additional live-recorded variables included latency to first vocalization and occurrence (yes/no) of vigilance behaviors in the 30 s following the first alarm call (visibly stretching neck, looking around, freezing, and erect posture) [25]. Each of the four vigilance behavior characteristics (erect posture, neck stretching, looking around, and freezing) were scored as either 0 (not observed) or 1 (observed), giving a vigilance score between 0 (no vigilance behavior observed) and 4 (all vigilance behaviors observed at least once) for each bird tested. Videos were used to record latency to first step from when the alarm call playback ended, as a potential additional indicator of anxiety to determine how long the birds remained in a motionless state after the alarm call playback [25,43].

### 2.5. Experiment 2—Attention Bias Test

After Experiment 1, the AB test was modified with an increased sample size, a group testing approach rather than testing individual birds, and allowing more time in the test arena if most (but not all) birds began feeding after the first alarm call was played. The AB test was performed on days 32, 33, and 38 of age with 12 randomly selected birds/pen (*n* = 144 birds across all pens) by two observers. These observers were trained by the researcher collecting data for Experiment 1. Inter-rater agreement was tested for latency to feed of 12 birds and was excellent among the three observers (Cronbach’s alpha of 0.933). The order of pens was randomized for testing. Birds were tested in groups of 3 (4 tests/pen) to avoid isolation stress [44]. The same location, arena, feeder, feed, and alarm call were used as described for Experiment 1 (Figure 3). Prior to placement in the arena, two out of three birds were marked with livestock marker (All-Weather Paintstik, LA-CO Industries, Inc., Elk Grove Village, IL, USA) for individual identification. Immediately after three birds were placed into the arena, the 8 s conspecific alarm call was played. Latency to begin feeding (s) from the feeder was then recorded for each individual bird (observer 1 recorded two birds, observer 2 recorded the third bird). Thereafter, the test procedure had four possible outcomes depending on how many birds began feeding and the time-point that they started feeding within the first 300 s of the test.

If all three birds fed from the feeder at least once during the 300 s testing period, they were allowed 5 s to feed before the second alarm call playback. Thereafter, the second alarm call was played. If all three birds fed from the feeder between 270–300 s, birds were allowed to feed for 5 s starting from when the last bird fed, the second alarm call was played, and the test time was extended to 420 s. Latency to resume feeding was recorded for each individual bird (observer 1 recorded two birds, observer 2 recorded the third bird). 

If at the end of the 300 s testing period, two out of three birds fed from the feeder, they were allowed 5 s to feed starting from when the last bird fed, then the second alarm call was played and the testing time was extended to 420 s. Latency to resume feeding was recorded for each individual bird (observer 1 recorded two birds, observer 2 recorded the third bird). The bird that did not feed received a maximum latency score of 300 s for latency to begin feeding and no score for latency to resume feeding.

If one of the tree birds fed from the feeder during the testing period, latency to begin feeding was recorded for the bird that began feeding, and the second alarm call was not played. The other two birds received a maximum latency score of 300 s.

If none of the three birds fed from the feeder during the testing period, all three birds received a maximum latency score of 300 s.

Video recordings were also used to record latency to step (s) and occurrence (yes/no) of vigilant behaviors within 30 s following the first alarm call. Each of the four vigilance behavior characteristics (erect posture, neck stretching, looking around, and freezing) were scored as either 0 (not observed) or 1 (observed), giving a vigilance score between 0 (no vigilance behavior observed) and 4 (all vigilance behaviors observed at least once) for each bird tested. It was not feasible to record latency to first vocalization because birds were tested in groups. 

### 2.6. Tonic Immobility Test

In both experiments, a single observer performed TI testing in the hallway area of the house, directly adjacent to the birds’ home pens. In Experiment 1, TI testing was performed on three randomly-marked birds/pen (*n* = 36) on day 12 of age. Birds were marked on their back with livestock marker (All-Weather Paintstik, LA-CO Industries, Inc., Elk Grove Village, IL, USA). The same marked birds were tested again on day 26 of age. In Experiment 2, TI testing was performed on six randomly selected birds/pen (*n* = 72) on day 19 of age. TI was induced by the handler carefully placing the bird on his back in a V-shaped cradle, placing one hand over the sternum and applying gentle pressure while cupping the other hand over the head (modified from [45]). After 15 s, the handler lifted her hands from the bird, moved out of the bird’s line of sight, and recorded latency until righting response (TI duration [s]). If the bird attempted to right himself within 10 s after the hands were lifted, TI was considered not induced and the handler repeated the restraint procedure (maximum of three induction attempts). If TI could not be induced, the bird received the minimum score of 0 s. If birds remained in TI for the full 300 s testing period, a maximum latency score of 300 s was given.

### 2.7. Statistical Analysis

Data were analyzed in JMP Pro 15 (SAS Institute Inc., Cary, NC, USA). Data residuals were assessed for their distribution by visual inspection of normal quantile plots. An overview of the distribution of data residuals and subsequent statistical approaches is shown in Table 3. The sample for resumption of feeding in the Experiment 1 AB test was too low for statistical analysis, so raw means are presented. For normally distributed data (see Table 3), with the exception of AB data in Experiment 2, general linear mixed-effects models were used, with complexity (HC/LC), stocking density (HD/LD), and their interaction as fixed effects, and pen as a random factor. For AB test data, age was not considered a factor, as treatment groups were randomized across testing days. Normally distributed AB data in Experiment 2 were analyzed using general linear mixed-effects models, with complexity (HC/LC), stocking density (HD/LD), and their interaction as fixed effects, and testing group nested within pen as a random factor. No significant interaction effect between complexity and density was found for any response variables, so the interaction term was removed from the models. Durations of TI in Experiment 1 were analyzed using general linear mixed-effects models with complexity (HC/LC), stocking density (HD/LD), day (bird age), day × complexity, and day × stocking density as fixed effects, with bird ID and pen as random factors. Tukey’s HSD test was used for post-hoc analysis when main factors or their interaction were significant at *p* < 0.05. Occurrence of vigilance behaviors were summed to give a total score, which ranged between 0 (no vigilance behavior observed) and 4 (all vigilance behaviors observed at least once), then were analyzed with complexity and stocking density as fixed effects, and pen as a random factor. Data are presented as LSmeans ± SEM unless otherwise noted.

## 3. Results

### 3.1. Experiment 1

#### 3.1.1. Attention Bias Test

Out of the 60 birds tested, 10 birds (4 from LC/LD, 3 from HC/HD, and 3 from HC/LD) began feeding after the first alarm call was played. No differences in latencies to begin feeding were found between either complexity (χ^2^ = 0.915; *p* = 0.339) or stocking density (χ^2^ = 1.715; *p* = 0.190) treatments (Table 4). Seven birds (2 from LC/LD, 2 from HC/HD, and 3 from HC/LD) resumed feeding after the second alarm call was played. No differences in latencies to resume feeding were found between either complexity (F_1,6_ = 0.528; *p* = 0.544) or stocking density (F_1,6_ = 0.892; *p* = 0.444) treatments (Table 4). No differences in latency to first vocalization were found between either complexity (F_1,59_ = 0.169; *p* = 0.691) or stocking density (F_1,59_ = 0.554; *p* = 0.476) treatments (Table 4). Latency to step did not differ between either complexity (F_1,44_ = 0.016; *p* = 0.904) or stocking density (F_1,44_ = 1.925; *p* = 0.215) treatments (Table 4). Looking around tended to be observed more frequently for birds from LD pens compared to birds from HD pens (χ^2^ = 3.298; *p* = 0.069; Table 5), with no other differences in frequency of observed individual vigilance behaviors between treatments. Vigilance behavior scores did not differ between either complexity (F_1,59_ = 0.062; *p* = 0.809) or stocking density (F_1,59_ = 1.552; *p* = 0.244) treatments (Table 5).

#### 3.1.2. Tonic Immobility Test

An interaction effect of environmental complexity and age was found for TI durations (F_1,35_ = 6.264; *p* = 0.015), with longer TI durations for birds from HC pens on day 12 compared to day 26 (*p* = 0.004; Table 6). No other pairwise differences were found (*p* > 0.12). Stocking density and age tended to impact TI durations (F_1,35_ = 3.15; *p* = 0.081), with birds from HD pens showing longer TI durations on day 12 than on day 26 (*p* = 0.016; Table 6). No other pairwise differences were found (*p* > 0.17). Attempts to induce TI did not differ on day 12 between either complexity (F_1,35_ = 1.03; *p* = 0.318) or stocking density (F_1,35_ = 0.041; *p* = 0.84) treatments, or on day 26 between either complexity (F_1,35_ = 1.287; *p* = 0.265) or stocking density (F_1,35_ = 0.463; *p* = 0.501) treatments (Table 6).

### 3.2. Experiment 2

#### 3.2.1. Attention Bias Test

**Figure 4:**
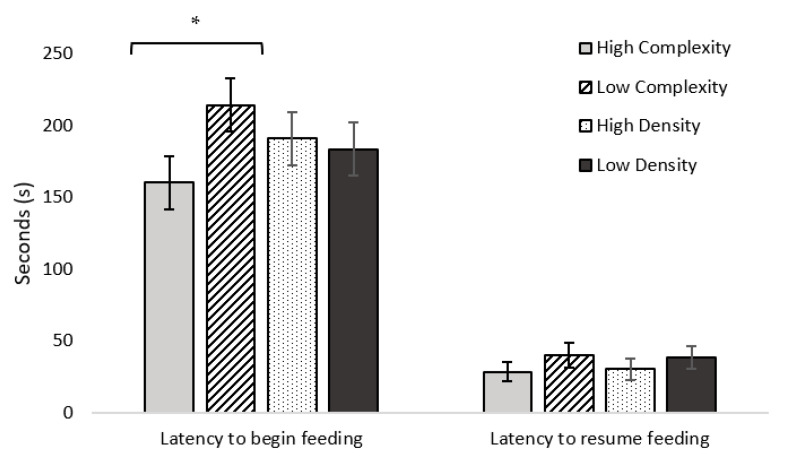
Least squares mean estimates (s ± SEM) for latency to begin feeding (*n* = 144) and resume feeding (*n* = 78) for broiler chickens kept in high-complexity, low-complexity, high-density, and low-density treatments in Experiment 2 at 4 and 5 weeks of age (days 32, 33, and 38). The timer was reset to zero after the second alarm call was played to record latency to resume feeding. * *p* < 0.05.

Out of the 144 birds tested, 92 began feeding following the first alarm call (19 from LC/LD, 21 from LC/HD, 24 from HC/HD, and 28 from HC/LD). Birds from HC pens began feeding faster than birds from LC pens (F_1,143_ = 4.430; *p* = 0.043; Figure 4). No differences in latency to begin feeding were found between stocking density treatments (F_1,143_ = 0.081; *p* = 0.777). Seventy-eight birds resumed feeding after the second alarm call was played (13 from LC/LD, 15 from LC/HD, 22 from HC/HD, and 28 from HC/LD). No differences in latency to resume feeding were found between either complexity (F_1,77_ = 2.658; *p* = 0.149) or stocking density (F_1,77_ = 2.413; *p* = 0.182) treatments (Figure 4). More birds from HC pens resumed feeding than birds from LC pens (50 from HC, 28 from LC; χ^2^ = 4.863; *p* = 0.027). No differences between stocking density treatments were found (χ^2^ = 2.109; *p* = 0.146; Figure 4). No differences in latency to first step were found between either complexity (F_1,99_ = 0.005; *p* = 0.946) or stocking density (F_1,99_ = 0.834; *p* = 0.368) treatments (HC: 101.55 ± 20.89 s; LC: 101.01 ± 20.82 s; HD: 114.51 ± 20.89 s; LD: 88.05 ± 20.82 s). Neck stretching behavior was observed more frequently in birds from LD pens than HD pens (χ^2^ = 4.559; *p* = 0.033), with no other differences in frequency of observed vigilance behavior between treatments. Vigilance behaviors scores did not differ between either complexity (F_1,98_ = 0.079; *p* = 0.780) or stocking density (F_1,98_ = 1.233; *p* = 0.275) treatment (Table 7).

#### 3.2.2. Tonic Immobility Test

**Figure 5:**
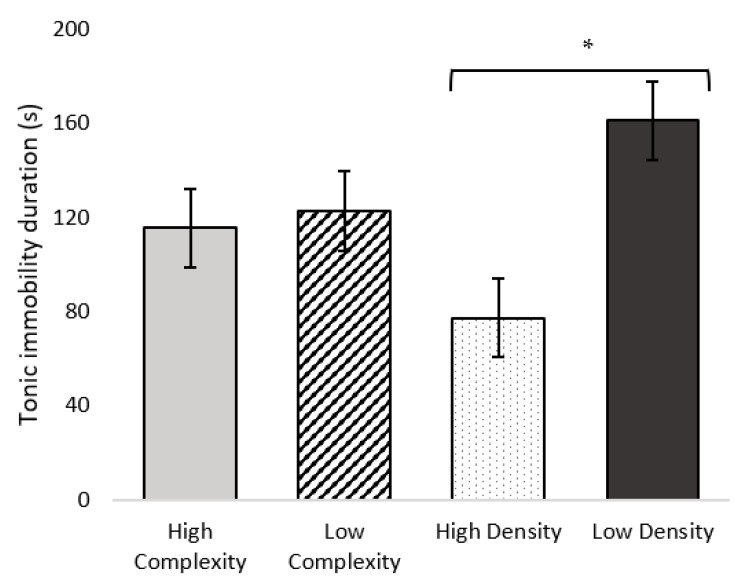
Least squares mean estimates (s ± SEM) for tonic immobility duration (0–300 s) for broiler chickens (*n* = 71) kept in high-complexity, low-complexity, high-density, and low-density treatments in Experiment 2 on day 19 of age. * *p* < 0.05.

There was no difference in TI duration between complexity treatments (F_1,70_ = 0.091; *p* = 0.770). Birds from HD pens had shorter TI durations than birds from LD pens (F_1,70_ = 12.610; *p* = 0.006; Figure 5). No differences in attempts to induce TI were found between either complexity (F_1,70_ = 1.016; *p* = 0.341) or stocking density (F_1,70_ = 0.074; *p* = 0.793) treatments. Mean TI induction attempts were 2.08 for HC, 1.86 for LC, 2.00 for HD, and 1.94 for LD pens (SEM of 0.15).

## 4. Discussion

This study investigated fear and anxiety in broiler chickens housed in either high or low environmental complexities and stocking densities. During the AB test in Experiment 1, birds from LD pens tended to look around more frequently than birds from HD pens, with no differences between the complexity treatments. Birds from HC and HD pens had longer TI durations on day 12 compared to day 26, whereas there was no difference for LC and LD birds. During the AB test in Experiment 2, birds from HC pens began feeding faster than birds from LC pens following the first alarm call playback, more birds from HC pens resumed feeding than birds from LC pens following the second alarm call playback, and birds from LD pens stretched their necks more frequently than birds from HD pens. These results suggest reduced anxiety in birds from HC pens compared to LC pens. Furthermore, birds from HD pens had shorter TI durations than birds from LD pens, indicating reduced fearfulness in birds from HD pens compared to LD pens.

### 4.1. Environmental Complexity

For the AB test, environmental complexity impacted latencies to begin feeding in Experiment 2, but not in Experiment 1. Longer latencies to begin feeding during a threatening situation suggests greater attention allocated towards the threat (alarm call), which indicates a higher level of anxiety. In Experiment 2, birds from HC pens were faster to begin feeding following an alarm call playback than birds from LC pens. This finding suggested reduced anxiousness in broilers housed in complex environments, which was in line with our hypothesis. Conversely, our results suggest that broilers housed in low-complexity environments biased their attention towards a perceived threat compared to a reward (feed). Therefore, these results link low-complexity environments to greater anxiety in broilers. By alleviating these negative states, high-complexity environments appear to improve broiler welfare. Attention bias tests performed with starlings [46] and laying hens [43] showed differences in level of anxiety in relation to environmental conditions or preference. Laying hens that preferred to remain indoors during the day responded more anxiously in an AB test compared to hens that preferred to go outside, observed through a small number of indoor-preferring hens eating during the test (only 7% of indoor-preferring hens resumed feeding after the alarm call playback compared to 36% of outdoor-preferring hens) [43]. Latencies to begin feeding in that study were comparable to those in the present study (indoor hens = 160 s vs. outdoor hens = 85 s compared to broilers from HC pens = 160 s vs. birds from LC pens = 214 s). Furthermore, our results do align with previous work in rodents that shows environmental complexity can reduce anxiety, although different behavioral tests were used in those studies, such as open field or elevated plus maze tests [47,48,49]. Ultimately, our AB results indicate that broilers housed in a complex environment are less anxious than those housed in a low-complexity environment.

Environmental complexity can decrease fear in broiler chickens, although some previous studies found no relationship. Access to elevated platforms resulted in shorter TI durations (238 s vs. 311 s) compared to access to manipulated standard resources (greater distance between feeders and water lines), suggesting reduced fearfulness in broilers housed with platforms [50]. These TI durations are longer than those observed in the current study, even though test approaches were comparable (LC: 123 s vs. HC: 116 s in Experiment 1). Broilers housed with perches and dust baths had shorter flight distances in an avoidance test, suggesting they were less fearful towards humans than control birds [28]. In Experiment 1, we found a difference in fearfulness within complexity treatments at different ages, but found no difference between complexity treatments. This is in agreement with other studies that did not report an impact of complexity on fear. For example, broilers housed with barrier perches did not have different TI durations compared to control birds [35,51]. Furthermore, responses during a novel object test to assess fearfulness did not differ between broilers housed with or without string enrichments [36]. Our results indicate that providing multiple enrichments concurrently did not impact fearfulness in broilers.

### 4.2. Stocking Density

Contrary to our predictions, stocking density did not affect birds’ responses during the AB test. In line with this finding, one previous study suggested that other housing conditions impact broiler welfare more than stocking density [52]. Stocking density can be especially influential later in life, with broiler welfare compromised when stocking densities are higher than 34–38 kg/m^2^, depending on final body weights [53]. Therefore, the potential detrimental effect of high stocking density could have been absent at the age that AB testing was performed, with high densities ranging between 19–21 kg/m^2^ (days 30, 32, and 33) in Experiment 1 and 25–30 kg/m^2^ (days 32, 33, and 38) in Experiment 2. We recommend that future research investigating the effect of stocking density on AB in broilers should perform the test later in life, when densities are at least 34 kg/m^2^.

We hypothesized that birds from HD pens would have longer TI durations and require fewer attempts to induce TI than birds from LD pens, indicating greater fear. However, in Experiment 1, we did not establish a difference between HD or LD treatments on TI durations, but there was a difference depending on age. This decrease in TI duration with age could reflect habituation to the test and handling, as the same birds were tested on both days. In Experiment 2, we found that birds in HD pens had shorter TI durations than birds in LD pens (HD: 72 s versus LD: 161 s in Experiment 2), suggesting birds from HD pens were less fearful compared to birds from LD pens. Past research suggests housing broilers at high stocking densities can contribute to increased fearfulness, which is contrary to our result. For example, broilers housed at a density of more than 18 to 22 birds/m^2^ had longer TI durations than broilers housed at lower densities [33,54,55]. Another study found that broilers housed at a high stocking density of 56 kg/m^2^ showed longer TI durations (more fearful) than broilers housed at lower densities [33]. Two of these lower stocking densities were comparable to the high and low densities at the time of TI testing in our study (6 kg/m^2^ and 15 kg/m^2^ compared to 8–16 kg/m^2^ and 4–8 kg/m^2^ at testing age in the present study), yet they did not find differences in TI duration between those two density levels (112 s for birds housed at 6 kg/m^2^ versus 101 s for birds housed at 15 kg/m^2^), whereas the present study found that birds from HD pens had shorter TI durations compared to birds from LD pens. The difference in results could be attributed to an age effect, as birds were tested for TI at 6 weeks of age in the previous study compared to 2- and 3 weeks of age in the present study. Broilers may be more fearful early in life, as young, small birds may perceive “safety in numbers” of greater importance than older, large birds. Domestic fowl have maintained pronounced anti-predator behavior, and so the value of being surrounded by many conspecifics is the reduced risk of predation and increased predator detection [56,57,58,59,60]. This could explain why birds in HD pens were less fearful than birds in LD pens at a young age. Contrary to our predictions and previous findings, birds from HD pens were less fearful than birds from LD pens. We recommend further research on this relationship.

### 4.3. Attention Bias Test Methodology

The AB test was modified after Experiment 1 to increase sample size and apply a group approach (three birds tested simultaneously) rather than testing individual birds. Broilers in Experiment 1 might have attempted to escape the testing arena faster due to social isolation, while in Experiment 2, broilers experienced social support from flock mates present, reducing their motivation to escape. In line, anecdotal observations did suggest social isolation distress based on the volume and pitch frequency of bird vocalizations and attempts to jump over arena walls in Experiment 1, but not 2. Broilers have a strong motivation for social reinstatement and chickens in natural settings live in relatively small, highly social groups [61,62,63,64]. Additionally, pairs of chicks placed in a novel open field test exhibited less fear-related behaviors than individual chicks in the same test [44]. Treatments did not impact latency to first vocalization in Experiment 1, latency to begin or resume feeding in Experiment 1, or vigilance behavior scores and latency to first step in both experiments. However, a large numeric difference between latencies to first step in Experiment 1 and 2 was found, with shorter latencies in Experiment 1 (27–49 s vs. 88–114 s). Therefore, latency to first step when birds are tested individually in a novel testing arena may indicate the birds’ motivation for social reinstatement rather than a measure of anxiousness.

The effects of environmental complexity and stocking density on attention bias in broiler chickens were previously unknown. AB tests were pharmacologically validated in laying hens—hens given anxiogenic drugs were slower to feed and faster to vocalize than hens receiving a saline injection, suggesting increased anxiousness in the former [25]. In our study, broilers’ latency to first vocalization (15–20 s) was much shorter than reported for laying hens, which vocalized after 114 s (control) and 317 s (hens that received an anxiogenic drug in Experiment 2 [25]). Similarly, latencies to first step in broilers was much shorter than (27–114 s) or comparable to previously reported results for laying hens (between 42–52 s and between 211–355 s [25]). Disparities in AB between broilers and laying hens could be due to different ages at the time of AB testing or genetic strain differences associated with selection for production traits [65,66,67]. Broilers have been genetically selected for fast growth rate [68], while laying hens were selected for traits associated with increased egg production [69]. Generally, it is accepted that different strains and breeds of domestic fowl possess different temperaments, most apparent in terms of fear or flightiness, which can be defined as rapid movement away from a stimulus [70,71,72,73]. Therefore, the temperamental differences between broilers and laying hens could explain the difference in responses seen in the AB test.

## 5. Conclusions

We investigated the effects of housing broiler chickens in a high- or low-complexity environment under high or low stocking densities on their level of fear and anxiety. The group approach to AB testing in Experiment 2 produced a difference in broiler responses between the complexity treatments, compared to the individual testing approach in Experiment 1. Broilers from high-complexity pens exhibited responses in the AB test suggestive of reduced anxiety compared to broilers from low-complexity pens, with no differences between the stocking density treatments. These results suggest that the environmental complexity provided in the present study improved welfare of broilers through reduced anxiety. To our knowledge, this is the first AB test successfully assessing anxiety in broiler chickens. Additionally, birds housed at higher stocking densities showed reduced TI durations, suggesting reduced fearfulness compared to birds housed at lower stocking densities. This finding counterintuitively indicates that, for broilers around 3 weeks old, housing at higher densities may reduce fearfulness.

## Figures and Tables

**Table 1:** Pairs of enrichment objects rotated every 3 days in high-complexity pens.

Nutritional Enrichment	Occupational Enrichment
Hanging bundles of white string	Free-moving metal ball (20.3 cm diameter) ^1^ filled with alfalfa hay
Yellow treat dispenser (7.6 cm diameter) ^2^ filled with whole-grain oats	Colored ball (5.8 cm diameter) ^3^
Laser light (5 min, 2×/day) ^4^	Experiment 1: Kong toy (5.6 cm diameter) ^5^ filled with iceberg lettuce
	Experiment 2: half a head of cabbage hung at bird height

^1^ Darice, Strongsville, OH, USA; ^2^ Lixit Corp., Napa, CA, USA; ^3^ Click N’ Play, USA; ^4^ Ethical Products, Inc., Bloomfield, NJ, USA; ^5^ KONG, Golden, CO, USA.

**Table 2:** Mean pen stocking density (kg/m^2^), and birds/m^2^, at day 1, 29, and 50 in Experiments 1 and 2.

Stocking Density	Experiment 1					
	Day 1		Day 29		Day 50	
	Kg/m^2^	Birds/m^2^	Kg/m^2^	Birds/m^2^	Kg/m^2^	Birds/m^2^
High	0.52	13.85	18.93	13.14	42.08	12.31
Low	0.26	6.92	9.81	6.71	23.83	6.29
**Stocking Density**	**Experiment 2**					
	**Day 1**		**Day 29**		**Day 50**	
	**Kg/m^2^**	**Birds/m^2^**	**Kg/m^2^**	**Birds/m^2^**	**Kg/m^2^**	**Birds/m^2^**
High	0.46	12.41	19.90	12.23	42.64	11.56
Low	0.23	6.21	10.22	5.97	23.31	5.79

**Table 3:** Summary of data analyses for Experiments 1 and 2.

Fear/Anxiety Test	Response Variable (Unit)	Distribution of Data Residuals	Statistical Approach
Attention bias	Latency to first vocalization (s) ^1^	Normal	General linear mixed-effects model
	Latency to first step (s)	Normal	General linear mixed-effects model
	Latency to begin feeding (s)	Other	Chi-square ^1^ and general linear mixed-effects model ^2^
	Latency to resume feeding (s)	Normal	General linear mixed-effects model
	Frequency to resume feeding (% of tested birds) ^2^	Other	Chi-square
	Vigilance behavior scores (0–4)	Normal	General linear mixed-effects model
	Frequency of vigilance behaviors	Other	Chi-square
Tonic immobility	Duration (s)	Normal	General linear mixed-effects model

^1^ In Experiment 1; ^2^ In Experiment 2.

**Table 4:** Least squares mean estimates (s ± SEM) for latency to first vocalization (*n* = 60), first step (*n* = 45), and begin feeding (*n* = 60), as well as raw means (s ± SEM) for latency to resume feeding (*n* = 7) for broiler chickens kept in high-complexity (HC), low-complexity (LC), high-density (HD), and low-density (LD) treatments in Experiment 1 at 4 weeks of age (days 30, 32, and 33).

Latencies (s)	Complexity Treatment		Stocking Density Treatment	
	HC	LC	HD	LD
First vocalization (s)	16.40 ± 5.35	19.51 ± 5.35	20.77 ± 5.35	15.14 ± 5.35
First step (s)	39.21 ± 9.58	37.09 ± 13.81	49.21 ± 10.62	27.09 ± 12.46
Begin feeding (s)	265.28 ± 13.95	296.80 ± 1.66	287.69 ± 7.13	274.39 ± 12.67
Resume feeding (s)	56.39 ± 42.24	22.73 ± 20.44	16.22 ± 9.87	58.99 ± 42.03

**Table 5:** Least squares mean estimates (± SEM) for vigilance behavior scores and % of total observations that each type of vigilance behavior was observed for broiler chickens kept in high-complexity (HC), low-complexity (LC), high-density (HD), and low-density (LD) treatments (*n* = 60) in Experiment 1 at 4 weeks of age (days 30, 32, and 33). Birds were scored either 0 (not observed) or 1 (observed) for each of four vigilance behavior characteristics (erect posture, neck stretching, looking around, and freezing), giving a vigilance score between 0 (no vigilance behavior observed) and 4 (all vigilance behaviors observed).

Indicators	Complexity Treatment		Stocking Density Treatment	
	HC	LC	HD	LD
Vigilance behavior score (0–4)	2.53 ± 0.19	2.47 ± 0.19	2.33 ± 0.19	2.67 ± 0.19
Erect posture (% of birds)	43.33	30.00	36.67	36.67
Neck stretching (% of birds)	50.00	53.33	46.67	56.67
Looking around (% of birds)	76.67	46.67	66.67 ^B^	86.67 ^A^
Freezing (% of birds)	83.33	56.67	83.33	86.67

^A–B^ Proportions with uncommon superscripts differ at *p* < 0.1.

**Table 6:** Least squares mean estimates for tonic immobility duration (s ± SEM; 0–300 s) and induction attempts (1–3) for broiler chickens kept in high-complexity (HC), low-complexity (LC), high-density (HD), and low-density (LD) treatments in Experiment 1 on days 12 and 26 (*n* = 36).

Measures	Bird Age (Day)	Complexity Treatment		Stocking Density Treatment	
		HC	LC	HD	LD
Tonic immobility duration (s)	12	109.43 ± 18.65 ^a^	51.24 ± 18.65 ^a,b^	101.42 ± 18.655 ^a^	59.25 ± 18.65 ^a,b^
	26	31.12 ± 18.65 ^b^	49.94 ± 18.65 ^a,b^	34.31 ± 18.65 ^b^	46.75 ± 18.65 ^a,b^
Tonic immobility induction attempt (1–3)	12	2.17 ± 0.19	1.89 ± 0.19	2.06 ± 0.19	2.00 ± 0.19
	26	2.39 ± 0.17	2.11 ± 0.17	2.17 ± 0.17	2.33 ± 0.17

^a,b^ Means with uncommon superscripts differ at *p* < 0.05.

**Table 7:** Least squares mean estimates (±SEM) for vigilance behavior scores and % of each type of vigilance behavior observed for broiler chickens kept in high-complexity (HC), low-complexity (LC), high-density (HD), and low-density (LD) treatments (*n* = 99) in Experiment 2 at 4 and 5 weeks of age (days 32, 33, and 38). Birds were scored either 0 (not observed) or 1 (observed) for each of four vigilance behavior characteristics (erect posture, neck stretching, freezing, and looking around), giving a vigilance score between 0 (no vigilance behavior observed) and 4 (all vigilance behaviors observed).

Indicators	Complexity Treatment		Stocking Density Treatment	
	HC	LC	HD	LD
Vigilance behavior score (0–4)	2.72 ± 0.15	2.66 ± 0.15	2.57 ± 0.15	2.80 ± 0.15
Erect poster (% of birds)	52.08	45.10	48.98	48.00
Neck stretching (% of birds)	66.67	56.87	51.02 ^b^	72.00 ^a^
Freezing (% of birds)	62.50	76.47	69.34	70.00
Looking around (% of birds)	89.58	88.24	87.76	90.00

^a,b^ Percentages with uncommon superscripts differ at *p* < 0.05.

## Data Availability

Data are available upon request from the corresponding author.
